# Improving the psychometric properties of the dissociative experiences scale (DES-II): a Rasch validation study

**DOI:** 10.1186/s12888-019-2417-8

**Published:** 2020-01-07

**Authors:** Aristide Saggino, Giorgia Molinengo, Guyonne Rogier, Carlo Garofalo, Barbara Loera, Marco Tommasi, Patrizia Velotti

**Affiliations:** 1 School of Medicine and Heath Sciences, University of Chieti – Pescara, Pescara, Italy; 2 Department of Psychology, University of Turin, Via Verdi 10, 10124 Turin, Italy; 3 Department of Dynamic and Clinical Psychology, University of Rome, Rome, Italy; 4 Department of Developmental Psychology, Tilburg University, Tilburg, Netherlands; 5 Department of Dinamic and Clinical Psychology, University of Rome, Rome, Italy

**Keywords:** Dissociative experiences, Item response theory, Category response rating, Offenders

## Abstract

**Background:**

The Dissociative Experiences Scale-II (DES-II) is a self-report questionnaire that measures dissociative experiences such as derealization, depersonalization, absorption and amnesia. The DES-II has been prevalently used as a screening tool in patients suffering from psychotic disorders or schizophrenia. However, dissociative experiences can also be part of normal psychological life. Despite its popularity, the most problematic aspect of the DES-II is the inconsistency in its factor structure, which is probably due to the tendency to treat ordinal responses as responses on an interval scale, as it is assumed in the Classical Test Theory approach. In order to address issues related to the inconsistency of previous results, the aim of the present study was to collect new psychometric evidence to improve the properties of the DES-II using Rasch analysis, i.e. analyzing the functioning of the response scale.

**Methods:**

Data were obtained on a sample composed by 320 Italian participants (122 inmates and 198 community-dwelling individuals) and were analyzed with the Rasch model. This model allows the estimation of participants’ level of dissociation, the degree of misfit of each item, the reliability of each item, and their measurement invariance. Moreover, Rasch estimation allows to determine the best response scale, in terms of response modalities number and their discriminant power.

**Results:**

Three items of the scale had strong misfit. After their deletion, the resulting scale was composed by 25 items, which had low levels of misfit and high reliability, and showed measurement invariance. Participants tended to select more often lower categories of the response scale.

**Conclusions:**

Results provided new knowledge on the DES-II structure and its psychometric properties, contributing to the understanding and measurement of the dissociation construct.

## Background

Dissociation is characterized by the alteration of those functions that normally allow an integration of the self, including identity, memory, consciousness, affectivity, perception, and cognition [1, 2]. When occasional, dissociative experiences are part of a normal psychological life in non-clinical populations. However, at a pathological level (in terms of frequency and associated distress), dissociation has been related with a wide range of psychiatric disorders [3–5]. Beyond psychiatric conditions, others maladaptive correlates have been linked to pathological dissociation, as for example violent behaviors [6, 7]. Consequently, the construct of dissociation appears to be a central aspect in psychiatry as well as clinical and forensic psychology [8, 9]. However, a consensual conceptualization of dissociation is still lacking [3]. For example, dissociation has been historically described as encompassing three domains, namely absorption, depersonalization/derealization and amnesia experiences [10], whereas another prominent conceptualization described two forms of dissociations, detachment and compartmentalization [11].

In the empirical literature, factor analytic studies on measures of dissociation attempted to clarify the underlying structure of dissociative experiences. Although new instruments have recently been developed, such as the Shutdown Dissociation Scale [12] and the Dissociative Symptom Scale [13], the Dissociative Experience Scale (DES) [2] and its revised version [10] remain the most widely used self-report instruments to measure the frequency of dissociative experiences [14], and it has been translated in several languages.

Unfortunately, studies exploring the factor structure of the DES-II yielded contrasting results, failing to provide consistent support for a specific conceptual model. Carlson and Putnam [10] provided initial evidence for a three-factor model, which was repeatedly found in some studies using exploratory (EFA) or confirmatory (CFA) factor analysis [6, 15–19]. However, using principal component analysis (PCA), Ray and colleagues [20] identified seven factors underlying the DES-II items. Also, using PCA, a four factor model was proposed by both Amdur and Liberzon’s [21] and Espírito Santo and Abreu’s [22] studies. Other studies found evidence of a two-factor solution, which was interpreted as distinguishing pathological and non-pathological dissociation using taxometric analysis [23]. A similar distinction between two forms of dissociation has also been found in two independent French samples (combining EFA and CFA) [24] and in a CFA study conducted by Armour et al. [25] in Northern Irish students. The distinction between pathological and non-pathological dissociation has also been replicated using eight of the DES-II items that are supposed to identify a ‘taxon’ of pathological dissociation [26]. The latter study differentiated the Absorption factor from a second one, encompassing Depersonalization, Derealization and Amnesia. Finally, among a sample of Italian inmates and community participants, a different two-factor model has been found employing EFA [27], supporting the description of two distinct, albeit correlated, dimensions of dissociation, namely detachment and compartmentalization [11]. Interestingly, such results partially converge with the three-factor solution found by Mazzotti et al. [28] in Italian clinical and non-clinical samples using CFA, with two of the factors reflecting detachment and compartmentalization.

As a whole, the inconsistency in the DES-II factor structure across studies and samples, as well as the high degree of shared variance among the factors, have led some authors to suggest that the instrument may actually capture a unidimensional operationalization of the dissociation construct [6, 8, 14, 26, 29–31]. Moreover, such contrasting results raise the possible risk of making misleading inferences about the construct of dissociation based on findings derived from the use of the original subscales reported by Carlson and Putnam [10] using the traditional Classical Test Theory (CTT) approach. Indeed, CTT often treats ordinal responses to a questionnaire items as intervals, possibly leading to erroneous conclusions and inferences about the scale under investigation, especially when a sum score is used to evaluate the degree to which an individual possesses a given characteristic [32].

Given such limitations, the aim of the present study was to examine the psychometric properties of the DES-II using Rasch analysis. Scales based on Rasch’s [33] approach to psychometrics fulfil the requirements of additive measurement [34]. Therefore, in the Rasch model, the sum score could be legitimately considered as a quantification of the construct being measured. According to Rasch’s approach, a person who has a greater ability than another person should have a greater probability of solving any test item. The probability of solving an easier item is greater than the probability of solving an harder item. The probability to answer properly to an item represents a function of two parameters: theta (subject’s ability) and beta (item difficulty). Rasch analysis assumes as a latent factor the probabilistic relationship between person ability and item difficulty, where the probability to answer correctly to an item is produced by the difference between a person’s ability and the item’s difficulty, with all items characterized by the same discriminationlevel. As such, the Rasch model locates a person’s ability and the item’s difficulty along the same continuum in logits, transforming ordinal data into interval-level measurement. Typically, such model is then compared with collected data in order to evaluate how close the actual results are to the predicted results. The closer the results are to the predicted results, the better is the fit of the data to the Rasch model. Unidimensional measures, fitting the Rasch model, are more appropriate for statistical analyses because differences between participants’ scores are interval-scaled and because the total score is an adequate representation of the dimension that is measured by the scale used.

The Rasch model was originally developed for dichotomous items and then extended to address every reasonable observational situation in the psychological and social sciences [35, 36]. Rasch analysis provides information that cannot be obtained using CTT approach [37]: it selects items in order to cover a wide range of the dimension being measured, and it is less sensitive to method factors (e.g., positively versus negatively formulated items) compared to confirmatory factor analysis (CFA) techniques [38]. The aim of the present paper was to propose a refined and more efficient version of the DES-II, based on the Rasch model, to be used in clinical settings.

## Methods

### Study design and participants

Data were collected using a self-administered questionnaire in a cross sectional study. The questionnaire included questions about background socio-demographic information and the DES-II scale. Community-dwelling participants were recruited through local advertisement posted online and throughout the community, requesting potential volunteers for psychological studies. A second group of participants was recruited in different jails and prisons located around two large Italian cities. Participants in this group were all incarcerated for having committed violent offenses. Each participant in the community sample completed the questionnaire individually. Participants in the incarcerated sample completed the questionnaire during small-group sessions settled in the prison library with the presence of a licensed psychologist.

The overall sample consisted of 320 participants: 122 were incarcerated individuals (age ranged from 21 to 77 years, M = 39.97 years, SD = 11.76) and 198 were community-dwelling participants (age ranged from 18 to 64 years, M = 32.51 years, SD = 10.30). All participants were Caucasian; 98% of incarcerated individuals and 58.6% of community-dwelling participants were males. For both groups, the following exclusion criteria were applied: cognitive disability and a diagnosis of psychiatric disorder. Four participants were removed due to missing data and consequently the study sample consisted of 316 cases.

#### Ethical considerations

The study received approval from the local university Ethical Review Board and the Italian Ministry of Justice (ERB Department of Dynamic and Clinical Psychology, Sapienza University of Rome, Protocol n. 10/2014). Participation was entirely voluntary, no payment was offered, answers were entirely anonymous and confidential, and there was no coercion for potential participants to take part in the study. All participants provided written informed consent to take part in the study. The study was conducted in conformity with the provisions of the Declaration of Helsinki in 1995 (as revised in Edinburgh 2000), and all ethical guidelines were followed as required for conducting human research, including adherence to the legal requirements of the country in which the study was conducted.

#### Measure

The Dissociative Experiences Scale-Revised (DES-II) [10] is a self-report scale that measures dissociative experiences in daily life related to depersonalization, derealization, amnesia, and absorption. The DES-II consists of 28 items. In the original DES, respondents were asked to indicate to what extent they experienced these symptoms (without being under the influence of alcohol or drugs) on 100-mm visual analogue scales. In the current DES-II, the analogue scales were replaced with a Likert-type scale ranging from 0%, meaning *never,* to 100%, meaning *always* (that is, containing 11 options at 10% increments). The total DES-II score is the mean of all 28 items scores. Previous research [10] has shown that the DES-II has high reliability (test-retest = 0.79 < *r* < 0.84; split-half = 0.83 < *r* < 0.93; Cronbach’s α = 0.95). Consistent with these findings, the Italian DES-II version [15] was equally reliable (Cronbach’s α = 0.91; split-half: *r* = 0.92). In the present study, we used the Italian translation reported by Conti [39], which showed excellent internal consistency (Cronbach’s α = 0.95) in previous research [27].

#### Statistical analyses

The Rasch model assumes unidimensionality. According to this assumption, a unidimensional model was applied to all of the 28 DES-II items. Whereas previous research revealed a two-factor structure of the scale [23, 25, 27], they reported high inter-factor correlations, supposing the possibility of a unidimensional construct. This would justify the use of a total score for measuring dissociation. Two types of Rasch models can be chosen to analyze polytomous items^1^: the rating scale model - RSM, [40] and the partial credit model – PCM [41]. The first model constrains all thresholds of responses to be identically distributed across all items, while the partial credit model do not specific such constraints on the thresholds.

Statistical analyses were performed on WINSTEPS 3.72.3 (Beaverton, Oregon). To assess the psychometric properties of the DES-II questionnaire, both PCM and RSM were estimated using a joint maximum likelihood method. Unidimensionality was tested by post-hoc principal component analysis of residuals and the critical value of eigenvalue ≤2 was chosen as the rule of thumb in the identification of a second dimension [42], whereas the correlation between residuals was used to check the assumption of local independence, considering *r*s < .30 as acceptable values. The INFIT and OUTFIT mean square statistics were used to investigate the degree of misfit of each item to the general domain. INFIT is sensitive to unexpected responses of persons with an ‘ability’ level near to the item difficulty, while outfit is sensitive to unexpected response observations distant from the item difficulty level. Ideal values for both are about 1.0 with the 0.5–1.5 range considered satisfactory [43]. Point-measure correlations (i.e., a measure of the correlation between single item scores and the Rasch measure) are reported considering positive values as acceptable.

We have considered also the person separation index (PSI), which indicates the spread of individual responses in standard error units. We then calculated strata using the formula: [(4PSI + 1)/3]. Strata are used to establish the number of statistically distinct levels of person’s ability that the items have distinguished [44]. Furthermore, the item estimate reliability (RI) shows how well the items that form the scale are discriminated by the sample of respondents. As suggested by Wright [45], good item separation is a necessary condition for effective measurement. In order to analyze whether the subjects used properly the response scale, category frequencies were considered firstly. Categories with frequencies ≤10 are described as problematic [42], because they do not provide enough observations for estimating stable threshold values. Moreover, category fit statistics as well as category probability curves were used as diagnostic tools. Lastly, a differential item functioning (DIF) analysis was performed to test measurement invariance. Despite different groups (e.g., incarcerated/community participants) being at equal levels of the underlying trait, they may respond to an item differently, indicating a bias between the groups. A difference of at least 0.5 logits between groups is noticeable, and indicates an item bias [46].

## Results

A descriptive analysis of the DES-II items is reported in Table 1.

**Table 1:** DES-II: Item descriptive statistics

	Mean	SD	Min	Max
DES-II1	18.5	21.4	0	90
DES-II2	29.4	22.1	0	100
DES-II3	11.0	18.4	0	90
DES-II4	5.7	15.3	0	90
DES-II5	9.0	16.9	0	100
DES-II6	11.6	18.4	0	100
DES-II7	8.9	18.4	0	90
DES-II8	3.8	13.0	0	90
DES-II9	7.0	15.4	0	90
DES-II10	17.1	21.9	0	100
DES-II11	6.4	16.3	0	100
DES-II12	9.2	19.6	0	100
DES-II13	6.7	17.8	0	100
DES-II14	26.7	25.7	0	100
DES-II15	17.8	22.7	0	100
DES-II16	13.4	20.1	0	100
DES-II17	21.6	25.8	0	100
DES-II18	14.9	21.4	0	100
DES-II19	12.3	22.6	0	100
DES-II20	17.1	23.7	0	100
DES-II21	21.1	27.8	0	100
DES-II22	12.2	20.3	0	100
DES-II23	25.1	26.8	0	100
DES-II24	20.4	23.4	0	100
DES-II25	11.9	19.5	0	100
DES-II26	11.2	20.2	0	100
DES-II27	7.2	19.4	0	100
DES-II28	6.0	16.5	0	100

Participants used the entire answer scale (0–100) for the majority of the items, with the exceptions of 6 items (DESII1, DESII3, DESII4, DESII7, DESII8, DESII9), for which the highest given answer was 90. However, the means of all items were low (ranging from 3.8 to 29.4) and the standard deviations were small (ranging from 13 to 27.8), indicating that participants frequently chose the lowest scale responses. The DES-II items adequately fitted only PCM specifications; post-hoc principal component analysis of residuals yielded a value of 2, while RSM showed a violation of the unidimensionality assumption, with the first eigenvalues of principal components analysis equal to 3.2. In Table 2, items are presented in order of misfit: 3 item (DES-II1, DES-II12, DES-II21) were deleted from the analysis because of marked deviations from the Rasch model expectations with INFIT and OUTFIT values outside of acceptable range. The PT-Measure correlation values were similar and positive for all items.

**Table 2:** DES-II: items misfit order, location and fit statistics (Partial Credit Model)

	LOCATION Logits	INFIT MNSQ	OUFIT MNSQ	PT-measure correlation
DES-II21	−.04	1.60	2.85	.38
DES-II1	−.02	1.54	1.67	.38
DES-II12	.02	.92	1.58	.36
DES-II10	−.02	1.07	1.48	.44
DES-II19	−.01	1.08	1.36	.38
DES-II17	−.03	1.28	1.35	.45
DES-II2	−.04	1.33	1.30	.50
DES-II23	−.04	1.22	1.17	.49
DES-II15	−.02	1.02	1.13	.45
DES-II18	.00	1.11	1.04	.43
DES-II28	.02	1.06	.55	.32
DES-II6	.02	.96	1.06	.40
DES-II14	−.04	1.03	.99	.53
DES-II26	.00	1.02	.93	.37
DES-II24	−.02	1.01	.99	.48
DES-II16	.01	.98	.95	.43
DES-II9	.02	.98	.82	.35
DES-II27	.02	.96	.63	.33
DES-II8	.04	.95	.64	.28
DES-II11	.03	.92	.47	.34
DES-II22	.01	.91	.77	.42
DES-II20	−.02	.89	.69	.47
DES-II25	.00	.81	.75	.42
DES-II13	.02	.79	.53	.34
DES-II5	.02	.78	.62	.40
DES-II3	.01	.75	.77	.43
DES-II4	.02	.69	.60	.34
DES-II7	.02	.63	.68	.40

*Note. DES-II* Dissociative Experiences Scale-Revise, *Outfit MNSQ* Outlier-sensitive fit statistic mean-square, Infit *MNSQ* Inlier-pattern-sensitive fit statistic mean-square, *PT*-measure correlation Point measure correlation

Tables 3 shows the misfit indices of the DES-II reduced to 25 items, along with location and fit statistics (PCM). The shortened DES-II version showed evidence of unidimensionality (first eigenvalue = 1.9) and the maximum correlation for the standardized residuals was 0.29. Thus, the local independence hypothesis was not violated. All of the INFIT and OUTFIT statistics were in the 0.5–1.5 satisfactory range.

**Table 3:** DES-II-25: items misfit order, location and fit statistics (Partial Credit Model)

	LOCATION Logits	INFIT MNSQ	OUFIT MNSQ	PT-measure correlation
DES-II17	−.03	1.39	1.50	.46
DES-II2	−.04	1.50	1.49	.51
DES-II10	−.02	1.11	1.46	.46
DES-II19	−.01	1.13	1.41	.40
DES-II23	−.05	1.29	1.21	.52
DES-II18	.00	1.21	1.13	.44
DES-II15	−.02	1.05	1.11	.48
DES-II14	−.05	1.10	1.05	.55
DES-II28	.02	1.09	.58	.33
DES-II6	.01	.98	1.08	.42
DES-II24	−.02	1.07	1.07	.50
DES-II26	.00	1.07	.99	.39
DES-II16	.01	1.01	.99	.45
DES-II9	.02	1.00	.90	.36
DES-II22	.00	.97	.87	.43
DES-II27	.02	.96	.90	.34
DES-II20	−.02	.96	.78	.49
DES-II8	.04	.95	.65	.28
DES-II11	.03	.92	.48	.35
DES-II3	.01	.81	.88	.44
DES-II13	.02	.84	.58	.34
DES-II25	.00	.82	.76	.43
DES-II5	.02	.79	.66	.41
DES-II7	.02	.65	.74	.40
DES-II4	.02	.69	.68	.34

*Note. DES-II* Dissociative Experiences Scale-Revised. *Outfit MNSQ* Outlier-sensitive fit statistic mean-square, *Infit MNSQ* Inlier-pattern-sensitive fit statistic mean-square, *PT*-measure correlation Point measure correlation

**Fig. 1:**
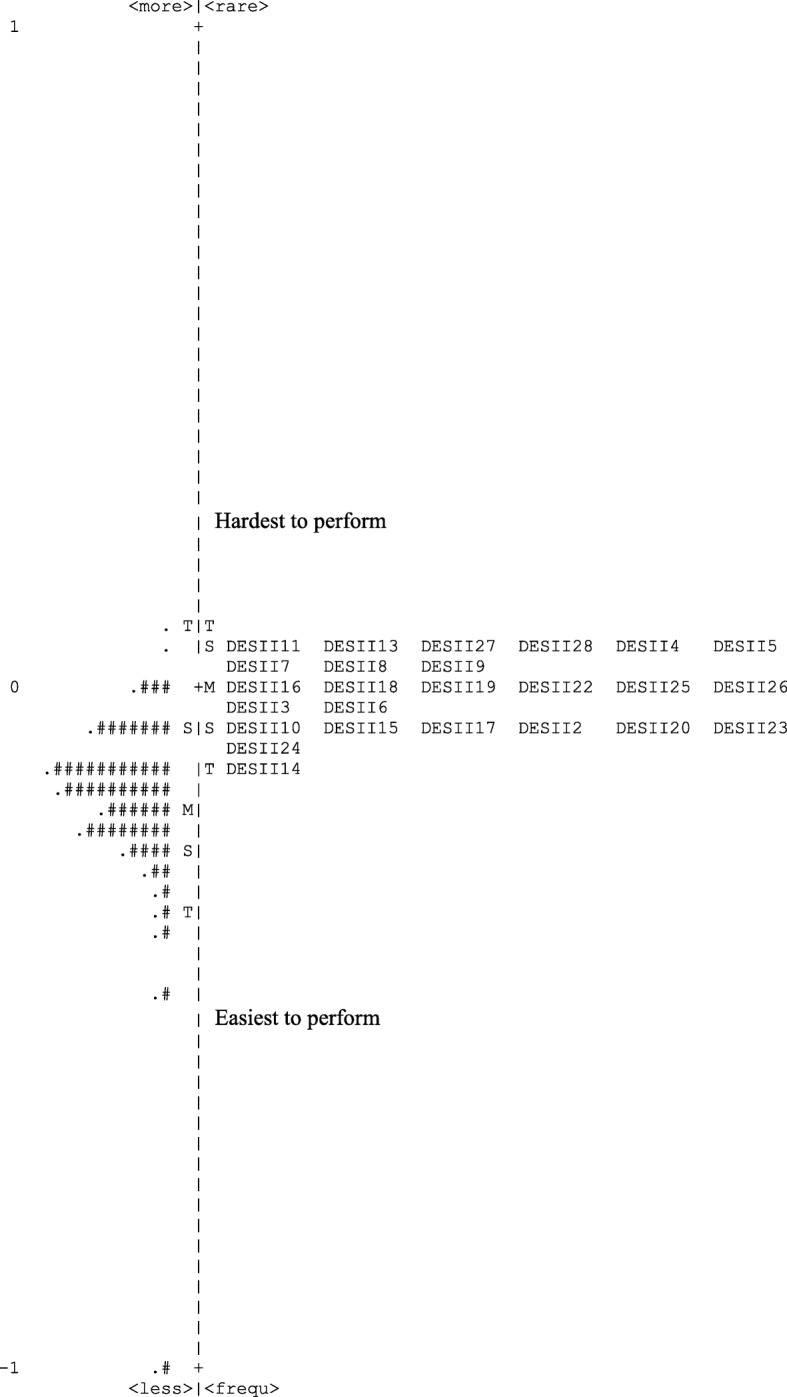
Logit map of all items and subjects. M = location of the mean measure; S = one standard deviation away from the mean measure; T = two standard deviations away from the mean measure

The DES-II 25 item version revealed satisfactory PSI and RI indices for both items and participants. The person reliability was high at 0.87 and the separation was 2.53. This separation indicates that the instrument identifies approximately four (3.71) statistically distinct strata of dissociation level. The item reliability was 0.97, indicating that the items were discriminated very well by respondents and the item separation was 5.63, indicating meaning that the spread of items was about 6 standard errors. The item locations along the logit scale (from easier to more difficult to rate) ranged from − 0.05 to + 0.04 logits. Inspection of the logit values (Fig. 1) revealed that the items were poorly distributed along the scale in terms of item difficulty, with no items covering the lower extreme of the continuum of the person’s level of dissociation, hence implying a floor effects. This indicates that the scale does not work well with subjects with a low scores of dissociation experiences.

**Fig. 2:**
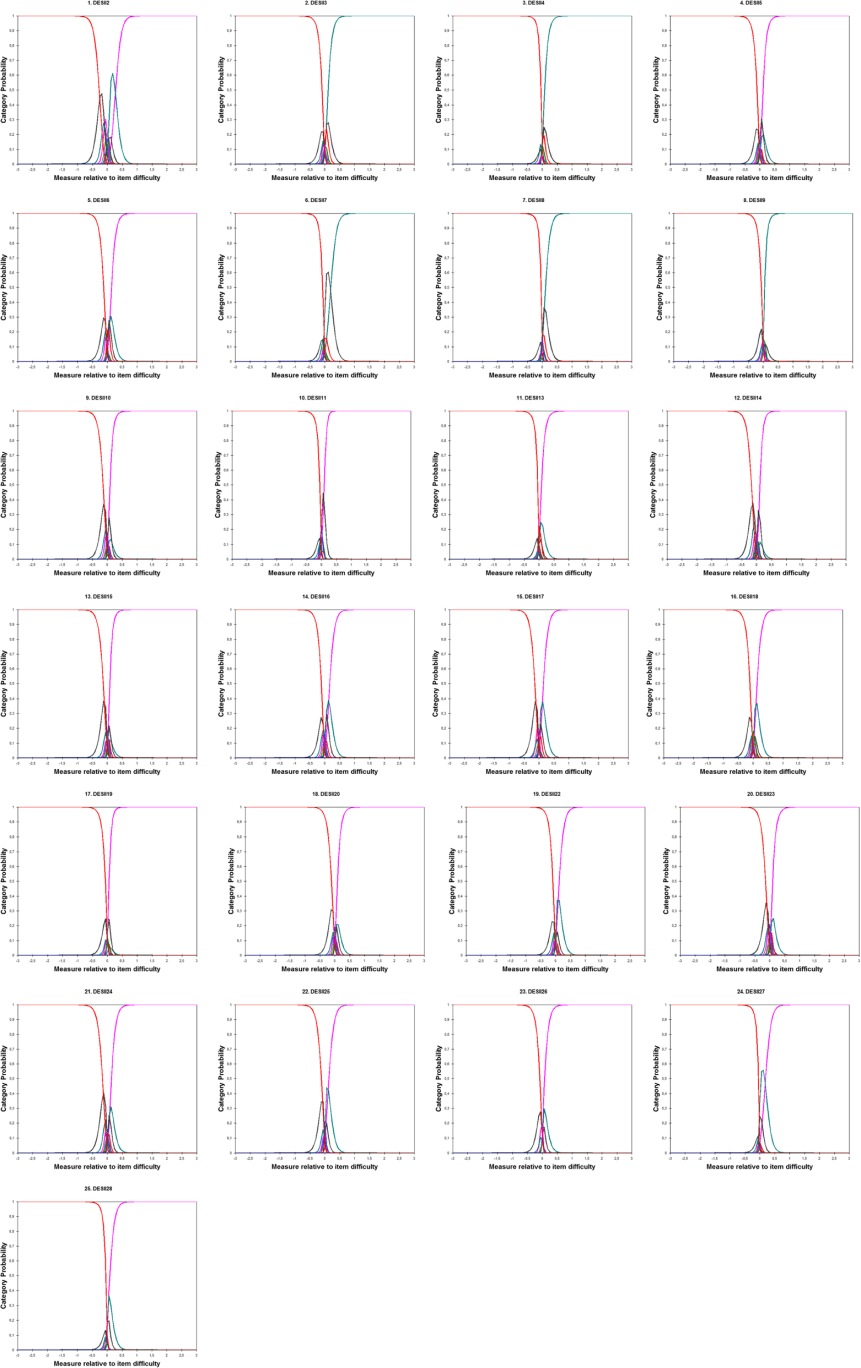
Category probability curves for all DES-II items

All 25 items had response categories with frequencies < 10, specifically the categories 60, 70, 80, 90, 100% never met the cut-off criteria. Moreover, the average measure did not ascend monotonically with category score as expected. Finally, in the inspection of category probability curves (Fig. 2), each category should have a distinct “top hill” in the curve, illustrating that each one has indeed a point at which becomes the most probable response category. In our case, extreme categories never emerged and most 3 and of others only peak for a very small range of the variable since the ideal number of response categories seem to be equal to 2 for all item. DIF analysis indicated that there was no differential item functioning between incarcerated and community-dwelling participants (DIF range = .00–.05), indicating that the DES-II works in the same way in the two groups by contrasting the response function for each item across the two groups.

## Discussion

The goal of the present study was to assess the psychometric properties of the DES-II, which have been previously analyzed only with the CTT approach, by applying Rasch analysis. To our knowledge, this was the first study that adopted the Rasch model to evaluate the psychometric properties of the DES-II. Rasch analysis can contribute to further our understanding of the dissociation construct, due to its specific psychometric characteristics, providing directions to develop a new Italian version of the DES-II based on results obtained with the Rasch model. Indeed, Rasch analysis allows to compare simultaneously item difficulty and persons’ ability on the same logit scale. This feature is of great importance and is not available following a CTT approach. The 11-point response categories of the DES-II could present severe problems, which were analyzed in depth by exploiting the features offered by the Rasch model [36, 47]. Specifically, results from this study highlighted that participants were unable to use and distinguish the extreme categories (i.e., 60, 70, 80, 90%).

Previous research has shown that participants’ style of responding has a strong effect in selecting response categories [48–50]. In particular, participants select categories not only on the basis of the intensity of their inner sensations or psychic processes and traits, but also on the basis of a strategy for a correct application of response categories to develop a valid judgment scale of the characteristic they have to evaluate [49, 50]. This strategy can lead participants to avoid the use of extreme categories, or to prefer lower or upper categories in their judgments [51]. Our findings suggested that participants in the present study did not use the highest categories to estimate their experiences of dissociation. Reasonably, this is due to the fact that our participants did not suffer from greatly impairing symptoms of dissociation, but it could also indicate that they tried to underreport the severity of their experiences in order to give a better image of their self (social desirability). Many studies showed that the optimal number of categories for a Likert scale is between 7 and 9, because scales are more reliable and less affected by biases in subjective responses [49, 51, 52]. However, the preference for a reduced set of categories can also affect the validity of a unidimensional scale. Lozano et al. [53] showed that a reduction of the number of categories reduced the explained variance of the latent factor, independently of the correlations between items.

Overall, the criteria for reliable measurement were met, but three item (DES-II 1, DES-II 12, and DES-II 21) were deleted from the analysis because of unsatisfactory INFIT and OUTFIT indices. These results were coherent with those of other studies that examined the DES-II items with different methods than factor analysis. For example, none of the deleted items were included in the DES-Taxon, the subset of items detected via taxometric analysis which is considered to address pathological dissociation [54]. Similarly, a correlation network analysis of the DES-II item scores showed that the centrality indexes of these three items were basically low, even though item 21 appeared to bear some relevance in the understanding of the dissociative symptom network [55]. The shortened 25-item DES-II version revealed a unidimensional construct, as indicated by a PCA of the residuals. From a clinical perspective, this allows psychologists and psychiatrists to confidently interpret sum scores as good indicators of individuals’ dissociation experiences.

However, in the present study a substantial floor effect was observed for the DES-II 25-item version, with the majority of participants actually reporting a very low level of dissociation experiences. Therefore, the DES-II may be more appropriate for more individuals with more severe impairment it is evident that there are no items targeting sub-clinical symptoms of dissociation [13].

The DIF approach within the framework of the Rasch measurement model offered a sophisticated way of confirming that incarcerated individuals and community participants responded in the same manner to all DES-II items. Our study shows the great value of Rasch analysis, which provides detailed item-level analysis and adds refinement to traditional psychometric methods [56–58]. In conclusion, we found that the DES-II performed well on most aspects of the assessment and the only serious problem for the DES-II seems to be the subjective strategy in the use of the 11-points response scale. Furthermore, three items did not work properly.

Overall, the unidimensional structure of the DES-II that emerged in the present study provided some support for the hypothesized interpretation of the inconsistent results obtained in previous factor analytic studies of the DES-II. That is, the different factor solutions, ranging from two to seven factors, that have been reported using Structural Equation Modeling approach may represent sample-specific variations rather than reflecting ‘true’ distinctions between conceptually separate factors. In addition, the facts that item-factor mapping varied across studies, and that inter-correlations among factors tended to be strong, are both consistent with the unidimensional structure of the DES-II reported in the present study. Our findings also suggest that the poor performance of certain items, based on Rasch analysis, could have influenced the identification of a stable factor structure in previous studies using the full DES-II scale.

A limitation of this study is that the results were obtained on an Italian sample only. Considering that validation of an instrument is a lengthy, even endless process [59] further studies across different countries should be performed to further test the psychometric properties of this tool. A further limitation is represented by the absence of a clinical sample, although the incarcerated sample was likely characterized by greater psychological problems than non-clinical samples. Therefore, future studies are needed to examine the replicability and generalizability of the present results in clinical populations.

## Conclusion

The novel application of the Rasch model to the study of the DES-II allowed us to provide new knowledge on the internal structure of this scale, in turn providing a contribution to the broader ongoing debate and increasing literature on the nature and structure of the dissociation construct. In conclusion, we propose that (a) the DES-II should be treated as a unidimensional index of dissociation, (b) items 1, 12, and 21 should be considered for deletion, and (c) the DES-II should be used with caution in non-clinical samples likely characterized by low levels of dissociation.

## Data Availability

The datasets used and/or analysed during the current study are available from the corresponding author on reasonable request.

## Abbreviations

CFA: Confirmatory factor analysis
CTT: Classical test theory
DES-II: Dissociative Experience Scale
DIF: Different item functioning
EFA: Exploratory factor analysis
PCA: Principal component analysis
PCM: Partial credit model
PSI: Person separation index
RI: Item estimation reliability
RSM: Rating scale model

## Footnotes

1 P(X_ni_ = x)= $\frac{\mathit{\exp}{\sum}_{k=0}^x\left[{\beta}_n-\left({\delta}_i-{\tau}_k\right)\right]}{\sum_{j=0}^m\mathit{\exp}{\sum}_{k=0}^x\left[{\beta}_n-\left({\delta}_i-{\tau}_k\right)\right]}$, x = 0,1,2,….,m where P(X_ni_ = x) is the probability that the individual n respond x to the question i; β_n_is the so called “ability” of the individual n (i.e. in this case the level of the latent trait that we want to measure), δ_i_ is the “difficulty” of the question (item) i (in practice how rare is to find an high score on this item),τ_k_ is the “difficulty” to reach level x = k; m is the maximum score.
